# A new milestone for *BioPsychoSocial Medicine*

**DOI:** 10.1186/s13030-018-0128-x

**Published:** 2018-08-06

**Authors:** Gen Komaki

**Affiliations:** International University of Health and Welfare, School of Health Sciences, Fukuoka, Japan

We are pleased to announce that *BioPsychoSocial Medicine (BPSM)* has been accepted by Clarivate Analyticsfor tacking in the Social Science Citation Index. In the recently- released 2018 Journal Citation Report, *BPSM* has received its first official *Impact Factor* of “1.0”.

It may be too early to tell what this first score would mean to this journal, or whether it fits or otherwise. We think more important thing is to keep the number growing from now onward, as the journal develops further.

Launched in 2007, the journal has grown significantly, with the number of published articles increased to more than 30 in 2017, or to the 10 years’ total of 256 articles. Seized its first impct factor as the journal’s new milestone, we are committed to strive to ensure further growth and development of *BPSM*.

*BioPsyhcoSocial Medicine* is the official journal of the Japanese Society of Psychosomatic Medicine. It plays an important role in providing the latest research in the field of psychosomatic medicine, especially focusing on mind-body relationships. It covers all aspects of the behavioral sciences, social sciences, neuroscience, stress physiology and epidemiology, psycho-neuro-endocrinology/ immunology, and psycho-oncology, all of which are associated with mind-body interactions [1].

**Fig. 1:**
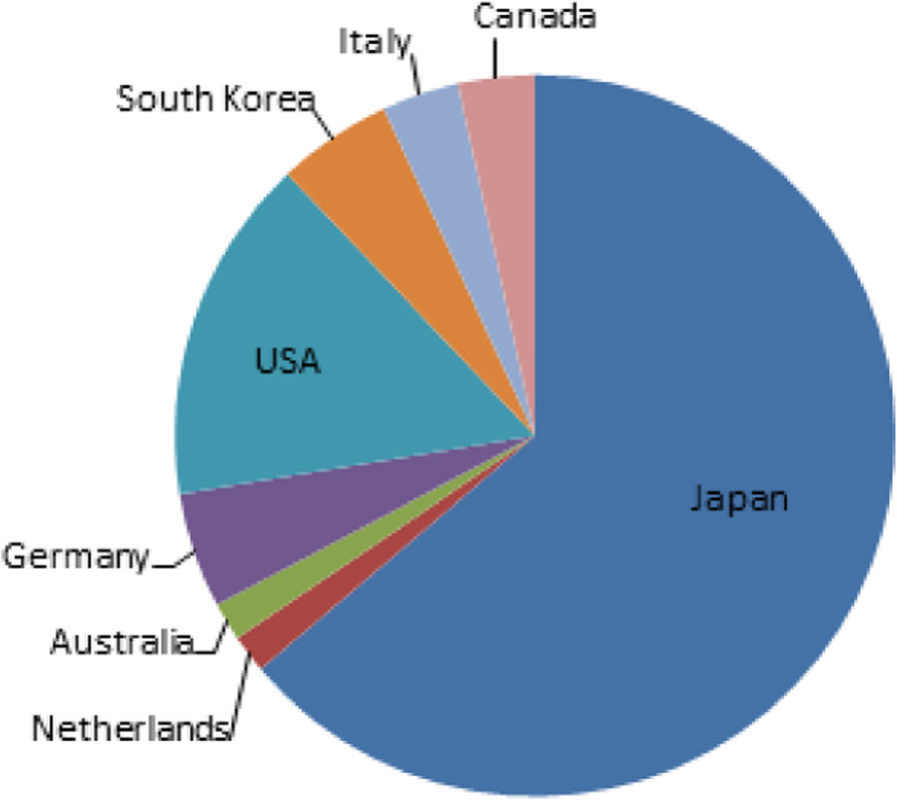
Geographical location of Editorial Board Members in 2017

The editorial board is comprised of a number of internationally well -respected experts representing a broad range of specialties and opinions (Fig. 1) [2]. The journal attracts readers from all over the world; in 2017, although over one third of our readers are from North America and another third from Asia and the Pacific (including Australia), visitors to the website were based in 180 different countries. The journal also has many readers in Europe, particularly Germany and the United Kingdom.

As an international journal with a wide audience, *BioPsychoSocial Medicine* receives submissions from a diverse range of authors, the largest subset of whom are from Asia- Pacific, due to the strong influence of the Japanese Society of Psychosomatic Medicine in that region. The journal also continues to attract submissions from Europe, Africa, and the Middle East. We seek to cover all aspects of the bio-psycho-social approach to illness and health and are proud to present a wide variety of research, particularly from the authors who may not ordinarily publish in English.

Moving into *BPSM*’s second stage of development, we earnestly welcome research articles from any countries throughout the world that discuss the cultural diversity of psychosomatic medicine and that integrate the approaches of oriental and western medicine, which will allow us to better understand the mind-body relationship and how best to support individuals with psychosomatic diseases.

Finally, we would like to thank our distinguished members of international Editorial Board for their efforts on behalf of the journal and our publisher, BioMed Central, for their in-house contribution to the speed and efficiency with which manuscripts are processed. Most importantly, we thank our authors and our hard-pressed reviewers.

Gen Komaki

Editor-in-Chief, *BioPsychoSocial Medicine*.
